# Combination Analysis of Ferroptosis and Immune Status Predicts Patients Survival in Breast Invasive Ductal Carcinoma

**DOI:** 10.3390/biom13010147

**Published:** 2023-01-11

**Authors:** Yang Yang, Dankun Luo, Wenqi Gao, Qiang Wang, Wenchao Yao, Dongbo Xue, Biao Ma

**Affiliations:** 1 Department of Biochemistry and Molecular Biology, Arnie Charbonneau Cancer Institute, Cumming School of Medicine, University of Calgary, 3330 Hospital Dr. NW, Calgary, AB T2N 4N1, Canada; 2 Department of Obstetrics and Gynecology, The First Affiliated Hospital of Harbin Medical University, Harbin 150001, China; 3 Department of General Surgery, The First Affiliated Hospital of Harbin Medical University, Harbin 150001, China

**Keywords:** breast cancer, ferroptosis, immune, prognosis, prediction

## Abstract

Ferroptosis is a new form of iron-dependent cell death and plays an important role during the occurrence and development of various tumors. Increasingly, evidence shows a convincing interaction between ferroptosis and tumor immunity, which affects cancer patients’ prognoses. These two processes cooperatively regulate different developmental stages of tumors and could be considered important tumor therapeutic targets. However, reliable prognostic markers screened based on the combination of ferroptosis and tumor immune status have not been well characterized. Here, we chose the ssGSEA and ESTIMATE algorithms to evaluate the ferroptosis and immune status of a TCGA breast invasive ductal carcinoma (IDC) cohort, which revealed their correlation characteristics as well as patients’ prognoses. The WGCNA algorithm was used to identify genes related to both ferroptosis and immunity. Univariate COX, LASSO regression, and multivariate Cox regression models were used to screen prognostic-related genes and construct prognostic risk models. Based on the ferroptosis and immune scores, the cohort was divided into three groups: a high-ferroptosis/low-immune group, a low-ferroptosis/high-immune group, and a mixed group. These three groups exhibited distinctive survival characteristics, as well as unique clinical phenotypes, immune characteristics, and activated signaling pathways. Among them, low-ferroptosis and high-immune statuses were favorable factors for the survival rates of patients. A total of 34 differentially expressed genes related to ferroptosis-immunity were identified among the three groups. After univariate, Lasso regression, and multivariate stepwise screening, two key prognostic genes (GNAI2, PSME1) were identified. Meanwhile, a risk prognosis model was constructed, which can predict the overall survival rate in the validation set. Lastly, we verified the importance of model genes in three independent GEO cohorts. In short, we constructed a prognostic model that assists in patient risk stratification based on ferroptosis-immune-related genes in IDC. This model helps assess patients’ prognoses and guide individualized treatment, which also further eelucidatesthe molecular mechanisms of IDC.

## 1. Introduction

Breast cancer is the most common cancer diagnosed among women worldwide and has become the leading cause of female cancer-related mortality [1]. Unfortunately, its increasing morbidity and mortality rates are ascertained which result in an inevitable financial burden not only for individual families but also for national medical systems [2]. Due to their heterogeneity, the prognosis for patients with different breast cancer subtypes varies [3]. The most common histological type of breast cancer is invasive ductal carcinoma (IDC), which accounts for 70–75% of total incidences [4,5], As the dominant category of breast cancer [6], IDC expresses lower hormone receptors than invasive lobular carcinoma(ILC), while exhibiting advanced blood vessel infiltration capacity [5] and a high percentage of the development of lung metastasis [7,8]. The disease specific survival(DSS) of IDC patients is significantly lower than those suffering from ILC [9]. Surgical removal is considered a current first-line treatment for breast cancer cases; however, recurring and distal metastases are frequently observed post operation. Therefore, the discovery of a novel biomarker is desired in order to establish more precise treatment plans for breast cancer patients.

Tumor immunotherapy, as an innovative treatment strategy in oncology, is recognized as an effective cancer treatment. However, the tumor environment and solid primary tumor attributes affect the outcomes of immune-related treatments. Additionally, resistance to immunotherapy occurs among subtypes largely due to breast cancer heterogeneity. It is undeniable that not all breast cancer patients would benefit from identical immunotherapy. Hence, combined immunotherapy approaches are the new trend in cancer treatment [10,11].

Ferroptosis, a new type of programmed cell death first proposed in 2012, is also known as iron-dependent oxidative cell death, which is morphologically and genetically different from apoptosis, necrosis, and autophagy [12]. Ferroptosis is characterized by iron accumulation and lipid peroxidation, where the phospholipid membrane undergoes iron-dependent oxidative modification, and the depletion of cysteine specifically triggers this form of cell death [13]. The pathophysiological mechanism of ferroptosis remains unknown. Nevertheless, it is closely related to various human diseases, such as ischemia-reperfusion injury [14], neurodegenerative disease [15], and different types of cancer, including breast cancer [16,17,18,19]. Interestingly, in an in-depth study of tumor immunity, it was found that ferroptosis also plays an important role in the regulation of tumor immunity. It is a double-edged sword in regulating the tumor immune microenvironment [20]. The occurrence of ferroptosis in different cells in the microenvironment will bring about different effects. Tumor cells that undergo ferroptosis can trigger anti-tumor immune responses. Meanwhile, activated CD8 T cells can mediate direct tumor cell killing by further inducing ferroptosis of tumor cells [21,22]. Thus, the induction of ferroptosis in tumor cells can trigger antitumor immunity and enhance the effect of immunotherapy. However, immune cells are equally susceptible or resistant to ferroptosis in different ways. When lipid ROS accumulates excessively in the tumor microenvironment, they trigger ferroptosis in cytotoxic T cells, leading to a decrease in T cell effector function and impaired antitumor immunity [21,23]. Tregs, which perform immunosuppressive functions, can rapidly induce GPX4 expression after TCR/CD28 co-stimulation activation to avoid ferroptosis [24]. Thus, balancing the dual role of ferroptosis in tumor cells, antitumor immune cells, and immunosuppressive cells is particularly important for anticancer therapy. Stratified analysis of ferroptosis and immune status in different patients may provide more precise therapeutic strategies in response to the heterogeneous characteristics of tumors.

In our study, we aimed to explore the interaction between cell ferroptosis and the immune status of patients, in addition to its influence on disease prognosis. Through combination analyses of IDC patients’ ferroptosis status and immune function data, we characterized a novel panel of ferroptosis-immune-related gene signatures and investigated their prognostic value as well as clinical characteristics among IDC patients.

## 2. Materials and Methods

### 2.1. Data Sources and Preparation

We obtained the IDC gene expression dataset and associated clinical information from public databases. Samples with insufficient clinical information were excluded, as well as cases with short survival time (<30 days) due to uncertainty of the cause of death. Gene expression data uniformed by FPKM from 660 IDC samples and 112 normal breast samples were downloaded from the TCGA database (https://portal.gdc.cancer.gov (accessed on 13 February 2021)) Three validation datasets (GSE21653; GSE45255; GSE61304) were chosen from the Gene Expression Omnibus (GEO) (https://www.geo.org/en/ (accessed on 22 March 2021)) Validation sequencing data were combined and corrected using the “sva” package. A total of 336 IDC samples with survival information were obtained as validation datasets.

BRAC mutation datasets were downloaded from TCGA and pre-processed by varScan2. R Bioconductor package “Maftools” were used to analyze and visualize Mutation Annotation Format (MAF) files, which calculated tumor sample mutation frequency and tumor mutation burden (TMB). The FerrDb database is the first validated ferroptosis function-related database (http://www.zhounan.org/ferrdb/index.html (accessed on 24 February 2021)). We accessed 256 ferroptosis-related genes, among them, 111 of which are ferroptosis markers, 108 are ferroptosis drivers, and 69 are ferroptosis inhibitors. There are also 28 genes annotated in multiple groups. Tumor immune infiltration was collected by multiple different algorithms on TIMER2.0 (http://timer.cistrome.org/ (accessed on 15 March 2021)). Those six algorithms were able to estimate the overall tumor immune infiltration status among TCGA samples [25]. The immunophenoscore was obtained from the TCIA database (https://tcia.at/ (accessed on 13 April 2021)) [26], which is a good predictor for evaluating the response of anti-cytotoxic T lymphocyte antigen 4 (CTLA-4) and anti-programmed cell death protein 1 (anti-PD- 1) in tumors. The TNMplot (https://www.tnmplot.com/ (accessed on 5 January 2022)) [27] platform was used for result validation, which organizes gene expression changes between tumors and normal and metastatic tissues from different databases. Proteomic data were obtained from the Human Protein Atlas database (https://www.proteinatlas.org/ (accessed on 12 August 2021)) [28] and the UALCAN platform (http://ualcan.path.uab.edu (accessed on 31 August 2021)) [29].

### 2.2. Tumor Ferroptosis Scores

Using the 256 ferroptosis-related genes obtained from the above database, we used the single-sample gene set enrichment analysis (ssGSEA) in the R package “GSVA” (R, conductor) [30] to calculate the gene set enrichment scores that were positively and negatively associated with the occurrence of ferroptosis (ES). The score obtained by normalizing the positive ES minus the negative ES is defined as the Ferroptosis score (FerrS) to evaluate the ferroptosis trend and level of the samples.

### 2.3. Tumor Immune Microenvironmental Analysis

Estimation of Stromal and Immune cells in Malignant Tumors using Expression data (ESTIMATE) algorithm was used to establish tumor environment component analysis. ESTIMATE algorithm generated immune scores using gene expression data which further predicted the proportion of basal and immune cells.

### 2.4. Differential Gene Expression Analysis

The “limma” package was used to extract DEG among different breast cancer subtypes (|logFC| ≥ 1.5, p.adjust < 0.05), and co-DEG was obtained after overlapping different groups.

### 2.5. Function Enrichment Analysis

We downloaded “clusterProfiler”, “org.Hs.eg.db”, and “enrichplot” packages from R conductor for functional enrichment analysis. Filter cutoff was set to PDF < 0.05 and followed by Gene Ontology (GO) biological processes and Kyoto Encyclopedia of Genes and Genomes (KEGG) Pathways analysis.

### 2.6. Gene Set Enrichment Analysis

R “enrichplot” package was used to differentiate enrichment pathways between high- and low-FerrS groups. Pathways were considered enriched if p.adjust < 0.05, q-values < 0.05, and (NES) > 1.

### 2.7. Weighted Correlation Network Analysis (WGCNA)

WGCNA analysis was performed to top 25% of variance genes using the “WGCNA” package [31], which aimed to find classification-related gene modules. The scale-free network was constructed with a SoftThreshold of 3. A motion hierarchical clustering tree was calculated using relative parameters (cutHeight = 500,000, minSize = 10, mergeCutHeight = 0.25), which assigned correlated genes into the same modules and calculated their relevance.

### 2.8. Signature Gene Identification and Risk Score Definition

We randomly classified 660 sample data into a training set and a validation set. Univariate cox regression analysis (*p* < 0.05) lasso regression analysis, and multivariate cox regression analysis were performed to evaluate the strength of its predictive survival in the training set analyzed by R packages (“glmnet”, “survival”, “survminer”). Only 2 of 34 genes (GNAI2, PSME1) could independently assess the survival prognosis of patients, and a dual-gene risk score (RS) model for survival prediction had been developed. Use the following formula: FIRS = (coef of GNAI2 × GNAI2 expression value) + (coef of PSME1 × PSME1 expression value).

### 2.9. Statistically Analysis

All statistical analysis was performed using RStudio and SPSS. The “limma” software package was used for differential expression analysis; “glmnet” and “survminer” software packages were used for Cox analysis and lasso regression analysis; “timeROC” was used for time-related ROC analysis and area under the curve (AUC) calculation; the Kaplan-Meier method was used for survival analysis; the Kruskal-Wallis test was used to assess the significance of differences in specific gene expression or immune cell components; the chi-square test was used to assess the correlation between typing and clinical traits; *p* < 0.05 was considered statistically significant (*p* < 0.05, “*”; *p* < 0.01, “**”; *p* < 0.001, “***”).

## 3. Results

### 3.1. IDC Ferroptosis and Immune-Landscape

**Figure 1:**
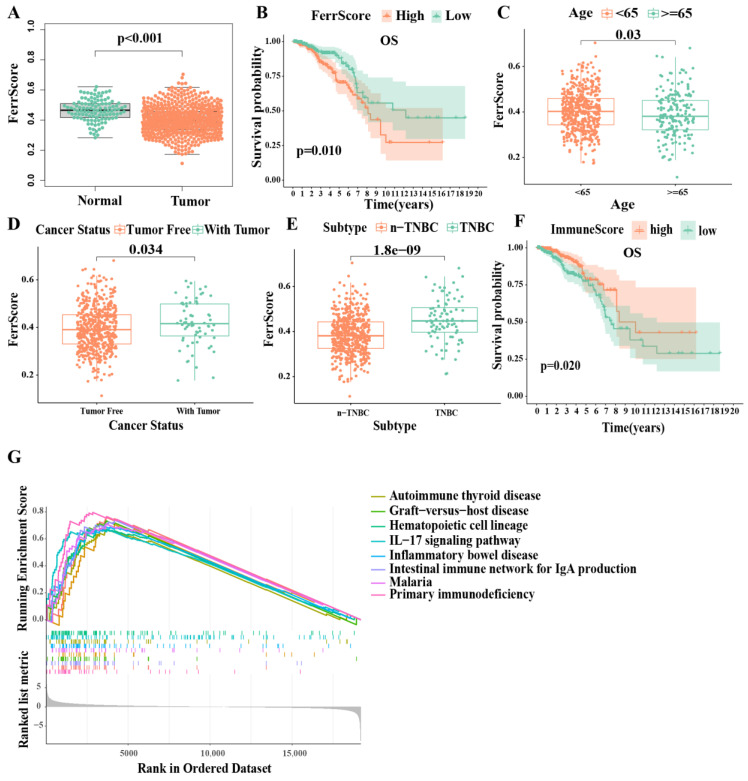
IDC ferroptosis and immune-landscape: The ssGSEA and ESTIMATE algorithms were used to evaluate the ferroptosis score (FerrS) and immune score (ImmS) in IDC samples. (**A**) FerrS in IDC and normal groups; (**B**) Kaplan–Meier overall survival curves for patients (n = 660) in high- and low−FerrS groups; (**C**–**E**) The difference of FerrS in clinical features; (**F**) Kaplan–Meier overall survival curves for patients (n = 660) in high- and low−ImmS groups; (**G**) GSEA KEGG enrichment analysis between high− and low−FerrS groups.

In order to investigate the levels of ferroptosis in IDC patients, we obtained 256 ferroptosis-related genes from the FerrDb database and calculated the ferroptosis score (FerrS) using the normalized positive core machinery components’ enrichment score (ES) minus the negative ES calculated by ssGSEA in IDC samples. The results showed that the FerrS of IDC patients was significantly lower than that of normal samples, which is consistent with a previous study (Figure 1A) [28]. As malignancies are often characterized by an iron-rich microenvironment to support rapid proliferation and progression, this creates an “addiction” of cancer cells to high iron levels, and it subjects tumors to persistent oxidative stress. Tumor cells thus become resistant to ferroptosis, thereby providing a growth advantage and leading to cancer chemoresistance. We believe that this may explain the lower iron death score in IDC. We then classified these IDC tumor data into two groups (high and low) based on the FerrS. A Kaplan–Meier survival curve indicated that high FerrS patients had an unfavorable prognosis in terms of their overall survival (OS) compared to the low FerrS group (Figure 1B). This is consistent with the fact that tumor cells possess stem-cell-like and dedifferentiated features that are more prone to ferroptosis [13]. We further explored possible factors that may interfere with the FerrS, including age, primary tumor status, and subtypes. The results showed that patients with an age <65 received a higher FerrS, as did patients with a recurrent tumor. Subtype analysis revealed that triple negative breast cancer (TNBC) subtypes lead to an advanced FerrS compared to non-TNBC patients, which may be due to the glutamine dependency of TNBC as the glutamine uptake or inhibition of system XC increased the level of intracellular ROS, leading to ferroptosis (Figure 1C–E) [32,33]. Subsequently, we performed gene set enrichment analysis on both the high- and low-FerrS groups. Gene ontology term analysis showed that genes in the high FerrS group were enriched in membrane disruption, T−helper 17 cell lineage commitment, etc. (Figure S1A) KEGG pathway analysis results revealed that DEGs with a high FerrS were highly associated with several cancer-development- and immune-signal-related pathways, such as the IL−17 signaling pathway, primary immunodeficiency, and the intestinal immune network for IgA production, suggesting that the ferroptosis score (FerrS) is able to predict the occurrence and development of tumors, as well as the modification of patients’ immune microenvironment (Figure 1G).

We then characterized the immune microenvironment of IDC patients by individually calculating their immune score (ImmS) by using the ESTIMATE algorithm. The ImmS ranged from −1116 to 3633 among all of the patients who were then further divided into two groups (high- and low-ImmS) based on the median value. Patients in the high-ImmS group had better prognoses than those in the low-ImmS group, indicating that immune function plays an important role in IDC patients (Figure 1F). Since the FerrS has an impact on immune signaling pathways, we analyzed the correlation between the FerrS and tumor immune infiltration. We obtained tumor infiltration results using six different algorithms from TIMER2.0 and analyzed their correlation with the FerrS. We can see that most immune promoting cells positively correlated with the ferroptosis score (FerrS, for example, the CD8+T, CD4+T, and NK cells. We believe that immune system activation was related to the release of damage-associated molecular pattern molecules (DAMPs) by ferroptotic cells [34]. Interestingly, immune suppressive cells such as Treg cells, showed an identical trend, indicating that a subset of cells that undergo ferroptosis may suppress the immune system and allow tumors to grow [13] (Figure S1B,C). Therefore, there is a complex regulatory network between ferroptosis and immunity, and it is necessary to stratify patients by taking both scores into account.

### 3.2. Ferroptosis and Immune-Related Subtypes Identification

**Figure 2:**
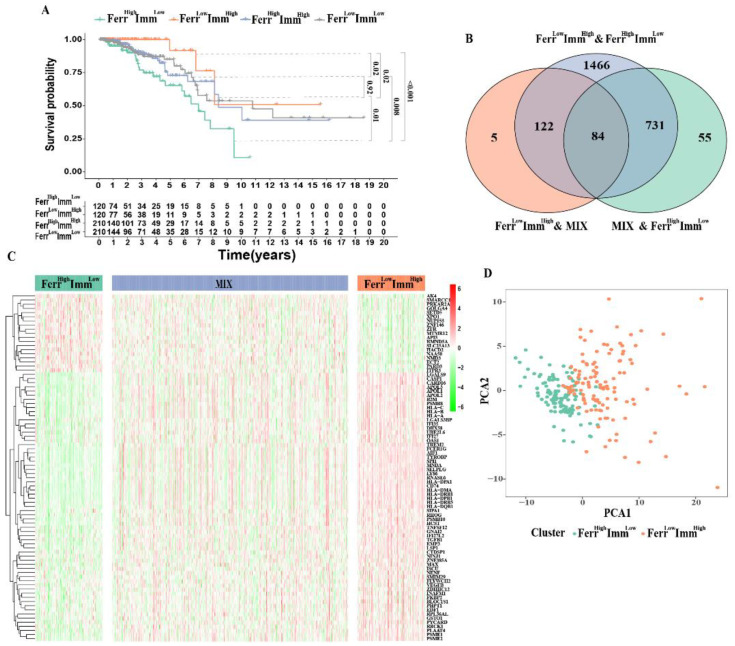
Ferroptosis and immune−related subtypes identification: (**A**) Kaplan–Meier survival curves for overall survival stratified according to the combined FerrS−ImmS signature. (**B**) The Venn diagram shows the number of DEGs among the three subtypes. (**C**) Heatmap of DEGs to visualize the expression levels among Ferr^High^Imm^Low^ (120), Ferr^Low^Imm^High^ (120) and MIX (420). (**D**) PCA based on the expression profile of DEGs according to Ferr^High^Imm^Low^ and Ferr^Low^Imm^High^ group.

Since the patients in the low-FerrS group and high-ImmS group have better prognoses, we screened the intersection patients of the two groups and named them the Ferr^Low^Imm^High^ group. Similarly, we screened out the patients in the high-FerrS group and the low-ImmS group with poor prognoses as the Ferr^High^Imm^Low^ group. The remaining patients were divided into the Ferr^High^Imm^High^ group and the Ferr^Low^Imm^Low^ group according to their scores. A survival analysis of the four groups produced expected results, with Ferr^Low^Imm^High^ patients having the best prognoses, Ferr^High^Imm^Low^ patients having the worst prognoses, and those of the Ferr^High^Imm^High^ and Ferr^Low^Imm^Low^ patients being in the middle. We also found that Ferr^Low^Imm^High^ patients showed a significant survival difference between Ferr^High^Imm^High^ or Ferr^Low^Imm^Low^ (*p* < 0.05), and Ferr^High^Imm^Low^ patients were the same (*p* < 0.05). However, was no significant survival difference between Ferr^High^Imm^High^ and Ferr^Low^Imm^Low^ patients (*p* = 0.92), suggesting that the FerrS and ImmS both had significant effects on IDC, and that the two may act as checks and balances. (Figure 2A). Considering that the molecular differences between groups with different prognoses may be an important site that affects the malignant behaviors of tumors, we combined Ferr^High^Imm^High^ and Ferr^Low^Imm^Low^ patients as the MIX group for a follow-up analysis.

Next, we retrieved 84 key DEGs (logFC > 1.5) associated with the FerrS and ImmS among the three groups (Figure 2B). The heatmap showed that most of the key genes were highly expressed in Ferr^Low^Imm^High^ patients, leading to a better survival rate (Figure 2C). Principal component analysis results exhibited a significant separation between the two clusters, Ferr^High^Imm^Low^ and Ferr^Low^Imm^High^patients, which clearly distinguished patients between the two groups for clustering (Figure 2D).

### 3.3. Clinical Features between Subtypes

**Figure 3:**
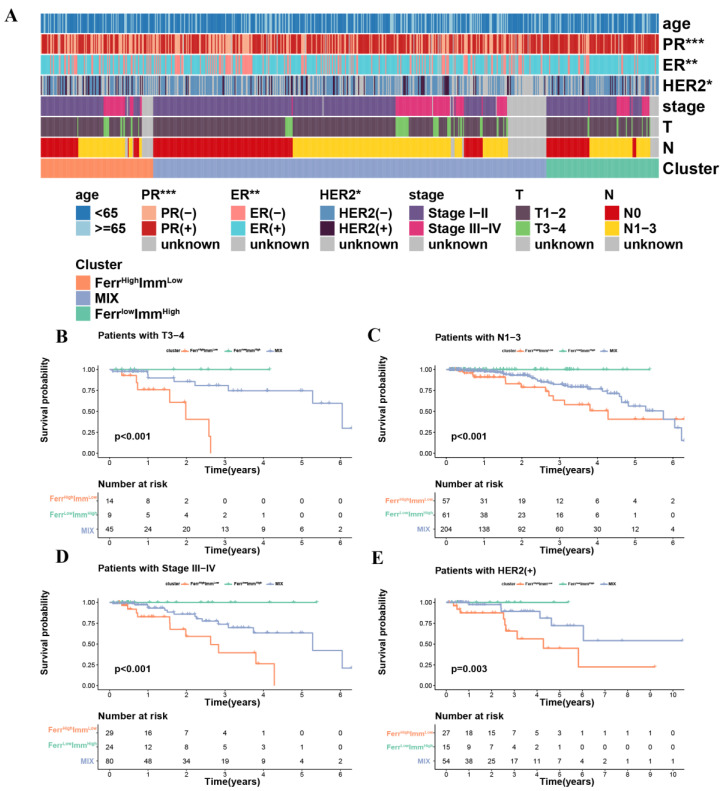
Differences in clinical characteristics and clinical stratified survival analysis among the three subtypes: (**A**) Heatmap of clinical features between subtypes. (**B**–**D**) The difference in OS between three groups stratified by stage. (**E**) The difference in OS between three groups stratified by HER2. (* *p* < 0.05; ** *p* < 0.01; *** *p* < 0.001).

In order to understand the differences in clinical characteristics among the three subtypes, we compared the age, tumor stage, TNM stage, ER, PR, and HER2 expression status data from the patients. Due to the small number of M1 stages in each subtype (<5), we did not discuss the differences in the M stages to ensure accuracy. Although there were no significant differences in the distribution of tumor staging among the three subtypes, significant differences in ER, PR, and HER2 expression were observed. (Figure 3A and Figure S2A–C) The hormone receptor expression level in the Ferr^High^Imm^Low^ group was relatively negative, and the HER2 expression level was positive. This suggested that such patients will be resistant to hormonal therapy. Trastuzumab has been widely used in HER2+ patients with satisfactory results, but the effect is mostly transient, and the enhanced oncogenic potential induced by HER2 cannot be underestimated, resulting in unsatisfactory clinical outcomes for patients [35]. Therefore, Ferr^High^Imm^Low^ patients may have rapid tumor progression and poor prognoses due to the limitation of treatment strategies. We further performed a stratified analysis of survival for each clinical feature to understand whether subtypes in different clinical statuses affect survival time. The results showed that the prognostic trend of each subtype in the stratified survival analysis of each clinical feature was consistent with the overall prognostic trend (Ferr^High^Imm^Low^ < MIX < Ferr^Low^Imm^High^). For the tumor stage, the subtypes had no significant effect on survival in the early stages (stages I and II), but differed significantly in the advanced stages (stages III and IV). This also suggested that being of the Ferr^High^Imm^Low^ group lead to rapid tumor progression, especially in advanced tumors. (Figure 3B–E and Figure S2).

### 3.4. Differences in the Immune Microenvironment among Subtypes

**Figure 4:**
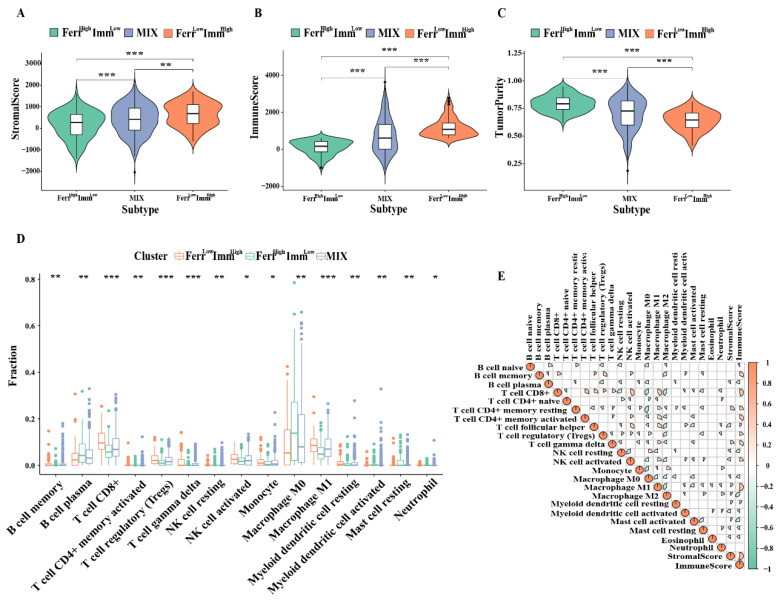
Distribution of immune microenvironment in subtypes: (**A**–**C**) Comparison of tumor microenvironment score (stromalscore, immunescore, and tumorscore) between three groups. (**D**) Comparison of infiltrating immune cells (CIBERSORT) between three groups. (**E**) Spearman correlation analysis was conducted to determine the correlation of immune cells and microenvironment. (* *p* < 0.05; ** *p* < 0.01; *** *p* < 0.001).

We further compared differences in the tumor microenvironment among the three subtypes. Patients in the Ferr^Low^Imm^High^ group had high immune score and matrix scores, whereas patients in the Ferr^High^Imm^Low^ group had high scores in tumor cells (Figure 4A–C). In addition, the tumor immune infiltration analysis also indicated that most of the anti-tumor immune cells, such as memory B cells, CD8+ T cells, activated NK cell, monocytes, and macrophage M1 were increased in the Ferr^Low^Imm^High^ group, whereas B cell plasma and macrophage M0 cells were observed in the Ferr^High^Imm^Low^ group (Figure 4D). The macrophage M0 and B cell plasma cells showed a strong negative correlation with most immune cells (Figure 4E). Interestingly, Tregs also showed high levels in Ferr^Low^Imm^High^ group. We consider that this may be related to the ability of Tregs to rapidly induce GPX4 expression after TCR/CD28 co-stimulation activation to avoid ferroptosis [24]. These results indicated that the proportion of immune cells infiltrated in the microenvironment changed among the subtypes, which led to differences in antitumor immunity among the groups.

### 3.5. Analysis of Ferroptosis and Immune-Related Genomic Heterogeneity among Subtypes

**Figure 5:**
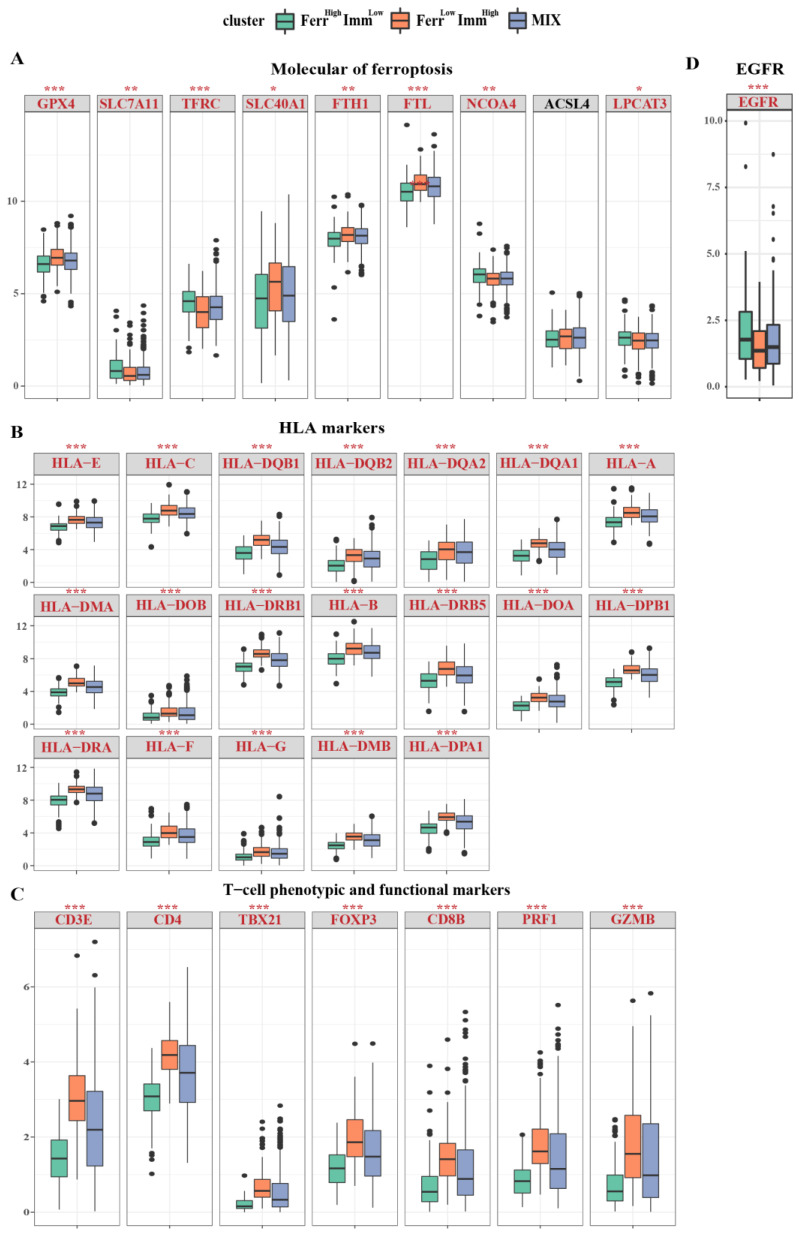
Distribution of individual expression of ferroptosis and immune−related genes among subtypes. Box and whisker plots show the expression of selected ferroptosis and immune-related genes between three groups. (**A**) The expression of ferroptosis−related genes between three groups; (**B**) The expression of HLA marker genes between three groups; (**C**) The expression of T cell phenotype and functional marker genes between three groups; (**D**) The EGFR expression between three groups; Data were analyzed using Kruskal–Wallis test; *p* values are reported as: * *p* < 0.05; ** *p* < 0.01; *** *p* < 0.001.

In order to explore the differences in the contribution of single-gene expression levels among each subtype, we analyzed and drew an expression map of significant genes among subtypes based on published key regulatory gene profiles of the ferroptosis pathway [36]. The results showed that a group of genes including GPX4, SLC40A1, and FTH1, was found to exhibit higher differential expression levels (Ferr^Low^Imm^High^ > MIX > Ferr^High^Imm^Low^), which are known as negative ferroptosis regulation factors. Meanwhile, genes that promote ferroptosis, such as TFRC, NCOA4, and LPCAT3, were elevated in the Ferr^High^Imm^Low^ group. These results validated the accuracy of FerrS in the judgment of ferroptosis. However, we found that SLC7A11 is low in the Ferr^Low^Imm^High^ group, which may be due to its complex function in IDC (Figure 5A).

Next, we employed the immune response-related gene signatures mentioned in the study of Zheng et al. [37] to compare their differences among the three subtypes. The results showed that the majority of key signature genes related to immunity displayed regular differential expressions, Ferr^High^Imm^Low^ < MIX < Ferr^Low^Imm^High^, which proved the various patterns of immune responses between groups. Overall, patients from the Ferr^High^Imm^Low^ group exhibited suppressive immune microenvironment features, other than Ferr^Low^Imm^High^ patients. Ferr^Low^Imm^High^ patients also present a higher expression level of human leukocyte antigens (Figure 5B). T cell phenotype and functional markers, as well as CD3E, CD4, TBX21, FOXP3, CD8B, PRF1, and GZMB, were elevated in the Ferr^Low^Imm^High^ group (Figure 5C). Except for the effector/repressor function markers (ARG1\NOS2), Ferr^Low^Imm^High^ patients presented with significantly increased expression levels in phenotypic and functional markers of myeloid lineage; Ferr^Low^Imm^High^ patients all exhibited significantly higher expression levels (Figure S3A). Other markers, including IFNγ markers (CXCL9, CXCL10, IDO1, IFMG, and STAT1) and immune modulators (ENTPD1) also showed higher expression levels in the Ferr^Low^Imm^High^ group (Figure S3B,C). However, the epidermal growth factor receptor (EGFR) related to tumor invasion and metastasis was expressed less in the Ferr^Low^Imm^High^ group (Figure 5D). In addition, we found that in the Ferr^Low^Imm^High^ group, immune activation receptors and immune suppressive receptors were highly expressed, indicating that this phenotype undergoes complex immune responses. (Figure S3D,E).

### 3.6. Relationship between Tumor Mutations in Different Subtypes

**Figure 6:**
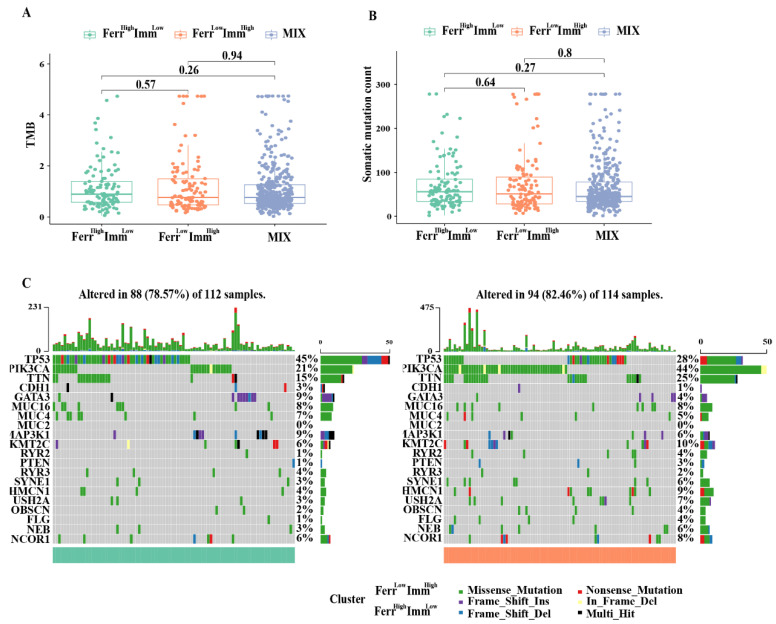
Relationship with tumor mutations in different subtypes: (**A**) Tumor mutational burden (TMB) of the three subtypes. (**B**) The number of mutated genes evaluated in the three subtypes (**C**). Frequency and type of mutations in the top 20 genes in Ferr^High^Imm^Low^ and Ferr^Low^Imm^High^ group. Genes are sorted according to frequency of mutations.

Previous studies have discovered that the tumor mutational burden (TMB) is closely related to the efficacy of immunotherapy. Therefore, we analyzed the variation in TMB and mutation frequency among different subtypes; however, there was no significance for TMB and mutation frequency between groups (Figure 6A,B). We also listed the top 20 most frequently mutated genes in both the Ferr^High^Imm^Low^ and Ferr^Low^Imm^High^ groups. As shown in Figure 6C, the gene mutation patterns were similar, and the tumor suppressor gene TP53 had the most significant mutation between the two groups. These findings indicated that the differences in clinical characteristics and prognoses between subtypes caused by gene mutations are not significant.

### 3.7. FerrS-ImmS Related Gene Screening and Functional Analysis

**Figure 7:**
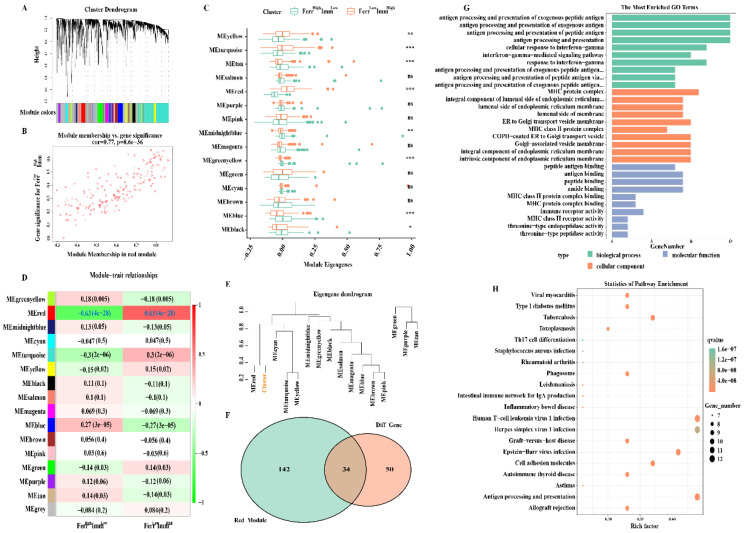
Ferroptosis−immunity related gene screening and functional analysis: (**A**) Gene co−expression network analysis. (**B**) Correlation scatter plot between the red module and the genes in the module. (**C**) Expression of the identified gene modules in the subtypes. (**D**) Diagram of the correlation between the module and Ferr^High^Imm^Low^ and Ferr^Low^Imm^High^ group. Each cell contains the corresponding correlation and *p* value. (**E**) Dendrogram of gene modules shows that the red module is highly correlated with the subtypes. (**F**) The Venn diagram shows the number of crucial genes among the DEGs and red module. (**G**,**H**) Gene ontology terms enriched and KEGG pathway analysis. ns, nonsignificant; * *p* < 0.05; ** *p* < 0.01; *** *p* < 0.001.

Weighted gene co-expression network analysis (WGCNA) is commonly used to identify gene modules that share relevant clinical traits or classifying characteristics. Therefore, we used the WGCNA method to screen out genes that are closely related to the Ferr^High^Imm^Low^ and Ferr^Low^Imm^High^ groups. First, we created a scale-free network (soft threshold = 3) and generated modules through motion hierarchical clustering tree cutting. After merging modules with similar heights, a total of 16 modules were generated. The “gray” modules contained genes that are not co-expressed and which were excluded from further analyses (Figure 7A).

We then analyzed the distribution difference of 15 modules between the Ferr^High^Imm^Low^ and Ferr^Low^Imm^High^ groups. Eight modules that were explored had significant differences. The Ferr^Low^Imm^High^ group exhibited higher intrinsic genes (ME) in the yellow, turquoise, and red modules, whereas they exhibited lower ME in the tan, midnightblue, greenyellow, blue, and black modules (Figure 7C). The correlation between modules and subtypes was then examined. It was found that the red module was significantly positively correlated with the Ferr^Low^Imm^High^ group (cor = 0.63, P = 4 × 10^−28^) (Figure 7D,E), and that the genes contained in this module were also highly correlated with the subtypes (cor = 0.77, P = 8.6 × 10^−36^) (Figure 7B). These genes may be key signature genes between the Ferr^High^Imm^Low^ and Ferr^Low^Imm^High^ groups which were selected for subsequent analyses.

Next, we overlapped the DEGs obtained above with genes from the red module, and acquired a total of 34 key genes (Figure 7F). Functional enrichment analysis revealed multiple biological processes that mainly involve antigen processing, antigen presentation, that is, gamma interferon reaction, etc. Cell components include the MHC protein complex, endoplasmic reticulum membrane composition, Golgi vesicles, etc. Molecular functions include antigen binding, immune receptor activity, and amide binding (Figure 7G). KEGG functional pathway analysis shows that genes are mainly enriched in immune-related pathways such as antigen processing and presentation, Th17 cell differentiation, cell adhesion molecules, etc. Additional pathways, such as the phagosome, have been found to directly or indirectly regulate iron accumulation or lipid peroxidation to coordinate the complex ferroptosis response [38]. In addition, studies have revealed there was also a high correlation between Th17 cell and lipid oxidation activity that could induce ferroptosis [39] (Figure 7H).

### 3.8. Risk Model of IDC Based on FerrS-ImmS Related Genes

**Figure 8:**
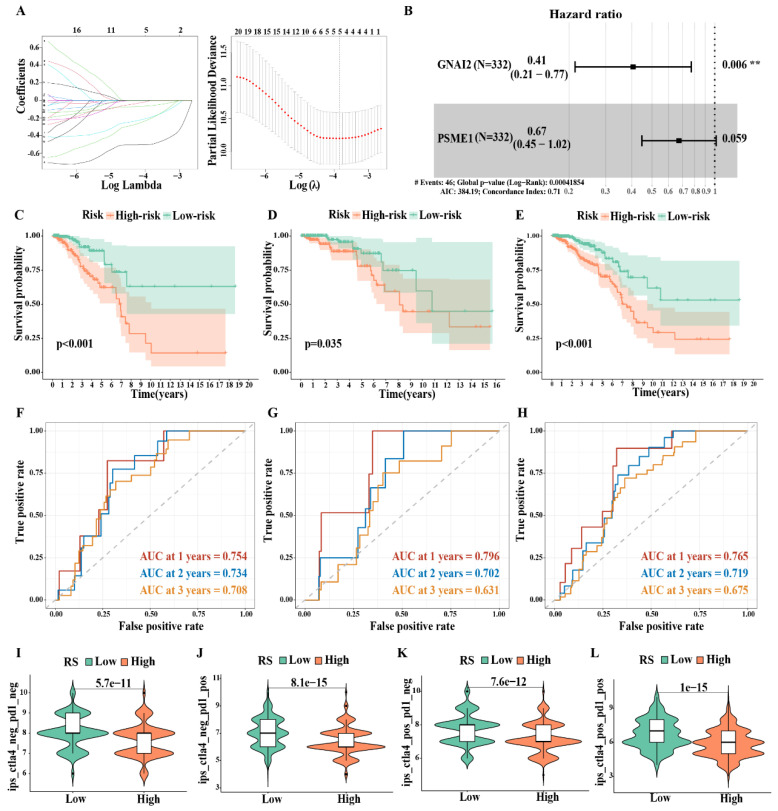
Risk model of IDC based on FerrS−ImmS related genes: (**A**) Lasso regression analysis. (**B**) Multivariate COX analysis. (**C**–**E**) Kaplan–Meier overall survival curves for patients in high− and low−risk groups of the Train group (**C**), Test group (**D**), and ALL group (**E**). (**F**–**H**) Time−dependent ROC curves at 1, 2, 3 years for patients in the Train group (**F**), Test group (**G**), and ALL group (**H**) to evaluate the prediction efficiency of the prognostic signature. (**I**–**L**) Immunotherapy sensitivity analysis between high− and low−risk groups. (** *p* < 0.01).

We randomly divided 660 samples into a training set and a validation set at a ratio of 1:1 (the clinical information is presented in Table S1). In the training set, a univariate Cox analysis (Table S2) and a Lasso cox regression model (Figure 8A) were used to evaluate the above 34 genes. These results confirmed that five genes have prognostic significance (*p* < 0.05). Next, we incorporated five genes into the multivariate COX regression analysis and adopted the stepwise regression method to determine the optimal risk score model: FIRS = (−0.90* GNAI2 expression value) + (−0.39* PSME1 expression value) (Figure 8B). A survival analysis showed that patients with a high FIRS had a more unfavorable prognosis than patients with low-FIRS (*p* < 0.001 in the Train group, P = 0.035 in the Test group, *p* < 0.001 in the ALL group) (Figure 8C–E). A time-dependent ROC curve analysis demonstrated the values of RS in the Train group (AUC of the first, second, and third years were 0.754, 0.734, and 0.708, respectively), the Test group (AUC of the first, second, and third years were 0.796, 0.702, and 0.631, respectively), and the ALL group (AUC of the first, second, and third years were 0.765, 0.719 and 0.675, respectively), which confirmed the accuracy and prognostic ability of FIRS among the three cohorts (Figure 8F–H).

We further compared the clinical differences between the high- and low-risk groups (Figure S4A). The results showed that there were significant differences in the expression of PR and ER between the two groups, whereas there were no significant differences in clinical stage. The Ferr^High^Imm^Low^ group was mainly distributed in the high-risk group and the Ferr^Low^Imm^High^ group was distributed in the low-risk group, which is consistent with our above analysis. In the univariate COX analysis, stage (*p* < 0.001), T (*p* < 0.001), N (*p* < 0.001), and FIRS (*p* = 0.002) were the prognostic factors affecting IDC patients. In the multivariate COX analysis, only stage (*p* = 0.007) and FIRS (*p* < 0.001) could be used as independent post-factors of IDC, and the risk index of FIRS was higher (Figure S4B,C). To further verify the accuracy of the FIRS model, we next compared three prognostic models, including four-gene signature (Ding) [40], five-gene signature (Du) [41], and seven-gene signature (Wu) [42] models with FIRS. The results showed that our model FIRS had a larger area under the curve (AUC) than the other models at 1, 2 and 3 years. Therefore, the FIRS model was determined in this study to be a more reasonable and efficient model, and fewer genes were used to determine the prognostic risk of IDC (Figure S4D–F).

Furthermore, we compared the sensitivity difference between the high-FIRS group and the low-FIRS group to immunotherapy in order to screen for the population that was the most suitable for tumor immunotherapy. They were further classified into four groups, namely, ips_ctla4_neg_pd1_neg (CTLA4-negative response and PD1-negative response), ips_ctla4_neg_pd1_pos (CTLA4-negative response and PD1-positive response), ips_ctla4_pos_pd1_neg (CTLA4-positive response and PD1-negative response), and ips_ctla4_pos_pd1_pos (CTLA4-positive response and PD1-positive response). Additionally, the results suggested that the population with a low FIRS had higher drug responsiveness, whether it was single immune checkpoint inhibitor administration or combined immunotherapy therapy, indicating the advantages of immunotherapy in these patients (Figure 8I–L).

### 3.9. Risk Model Gene Expression and Prognostic Characteristics

**Figure 9:**
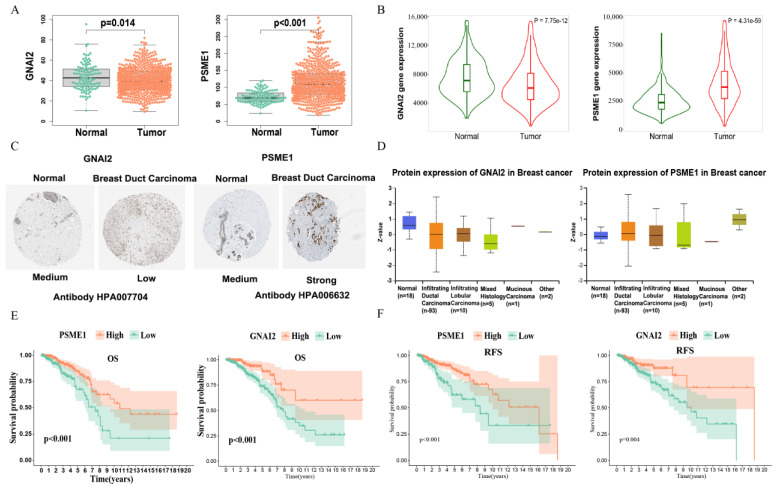
Risk model gene expression and prognostic characteristics: (**A**) Differential expression of risk model gene between tumor tissues and normal tissues in TCGA. (**B**) Differential expression of risk model gene between tumor tissues and normal tissues in TNMplot platform, which contains multiple database transcriptome data. (**C**) Differences in protein expression of the risk model gene in tumor tissue and normal tissue from Human Protein Atlas immunohistochemistry. (**D**) Differences in protein expression of the risk model gene in tumor tissue and normal tissue from the UALCAN platform. (**E**) Kaplan–Meier overall survival curves for patients in high− and low−expression groups of risk model gene of the TCGA cohort. (**F**) Kaplan–Meier relapse−free survival curves for patients in high− and low−expression groups of risk model gene of the GEO cohort.

To investigate the clinical significance of prognostic marker genes, we compared the differential expression of prognostic marker genes in normal tissues and IDC tissues. The results showed that G Protein Subunit Alpha I2 (GNAI2) was elevated in tumor samples at the mRNA level, whereas the PSME1 mRNA expression level was significantly reduced in tumor tissues (Figure 9A). We verified through the TNMplot platform [27], and obtained similar results (Figure 9B). Due to post-transcriptional regulation or translational regulation, such as protein degradation, gene expression may not be related to final protein expression. Therefore, we studied the protein levels of marker genes between normal breast tissue and IDC samples using data from the Human Protein Atlas database [28], and found that protein expression levels were consistent with mRNA expression results (Figure 9C). The UALCAN platform [29] collected the proteomics results in the CPTAC database; we verified them through the platform and received consistent results (Figure 9D). A survival analysis showed that samples that had highly expressed GNAI2 and PSME1 had better prognoses in OS in the TCGA database (Figure 9E). In addition, eligible corrected data from the GEO database underwent a combined survival analysis. The results also showed that the highly expressed PSME1 and GNAI2 had better RFS, which verified the importance of PSME1 and GNAI2 in IDC (Figure 9F).

## 4. Discussion

The heterogeneous state of breast tumors often results in a complex immune microenvironment, and patients with a “hot” immune microenvironment often have better prognoses. This difference in survival is often based on the abnormal molecular features rather than tumor tissue type. Therefore, more and more studies have attempted to identify subgroups based on molecular profiles of tumor patients to reflect different treatment responses and prognoses among patients. Ferroptosis is a new iron-dependent cell death mode discovered in recent years that can effectively inhibit the growth and development of tumors by inducing ferroptosis [43]. Interestingly, tumors with different differentiation states have different sensitivities to ferroptosis. Tumor cells with tumor stem-like features as well as dedifferentiated features are more prone to ferroptosis [13]. This implies that the induction of ferroptosis may be a promising therapeutic strategy for this class of highly progressive tumor subtypes. This feature was also confirmed in our study; for example, TNBC subtypes with poor prognoses in breast cancer classification had a higher FerrS than non-TNBC subtypes did. Considering the fact that IDC patients with different ferroptosis and immune statuses have different prognoses, there is a complex regulatory network between the occurrence of ferroptosis and immune responses [13,44,45]. Therefore, in this study, we divided the samples into four different subtypes according to the FerrS and ImmS, which showed different survival characteristics. Among them, the survival difference between the Ferr^Low^Imm^High^ group and the Ferr^High^Imm^Low^ group was the most obvious, and these two subtypes have unique clinical phenotypes, immune characteristics and activated pathways. Furthermore, based on these two subtypes, we constructed a risk model of RS that can be used to predict patient outcomes, which can be used as an independent prognostic factor for IDC. Further immunotherapy predictions also suggest that RS may be a promising immunotherapy stratification marker. These results facilitate the clinical management and precision treatment of patients.

In the analysis of the clinical characteristics of the three subtypes, we found significant differences in hormone receptor expression as well as HER2 expression among the three subtypes, with the Ferr^High^Imm^Low^ group exhibiting lower hormone receptor expression and higher HER2 receptor expression. This implies that the use of conventional hormone receptor-targeting drugs may be clinically limited in such patients. In addition, high HER2 expression would lead to more aggressive tumor growth [35], which corroborates the worse prognosis of the Ferr^High^Imm^Low^ group in patients with advanced stages (stages III and IV).

We further characterize the molecular characterization of the Ferr^Low^Imm^High^ and Ferr^High^Imm^Low^ groups in ferroptosis and immunity. Ferroptosis key signature genes were highly expressed in the Ferr^High^Imm^Low^ group, indicating the heterogeneity of ferroptosis in IDC patients. In terms of immune infiltration, key anti-tumor immune cells, such as T cell CD8+, activated NK cells, and macrophage M1, have been observed to be a dominant proportion in the Ferr^Low^Imm^High^ group, the same being true for immune-related characteristic genes. Interestingly, it seems contradictive that both activated and inhibitory immune markers are elevated in the Ferr^Low^Imm^High^ group. This result may imply the exhaustion of an anti-tumor immune response which leads to a gradually increased tumor immune response. Therefore, patients in the Ferr^Low^Imm^High^ group can be selected and suitable for immunotherapy because of an immune checkpoint blockade and the restoration of an anti-tumor immune response [46].

Due to the significant differences in clinical outcomes among subtypes, we aimed to explore the molecular mechanisms behind these groups. We screened out the genes that were most relevant to the different subtypes using the WGCNA method and then used a Cox analysis to finally construct a prognostic model containing two genes (PSME1 and GNAI2). G Protein Subunit Alpha I2 (GNAI2), as a G protein family member, is a vital signal transduction molecule. It suppresses the activity of adenylate cyclase (AC), thereby reducing cAMP content in the cell, which is further involved in a series of biological processes. cAMP signaling is closely related to mitochondrial function. Inhibition of Epac1, an effector of cAMP, prevents the onset of cellular ferroptosis induced by erastin and the loss of mitochondrial integrity caused by ferroptosis [47]. GNAI2 exerts different biological functions in different cancer types. For example, its high expression promotes the development and progression of colitis-associated, gastric cancers, and ovarian cancers [48,49,50]; but it inhibits the proliferation of squamous cell carcinoma of the tongue and hepatocellular carcinoma. In addition, GNAI2 is involved in the shaping of the immune microenvironment. Yu et al. [48] found that GNAI2 induces a “hot” immune microenvironment in gastric cancer, which is positively correlated with a variety of chemokines that promote cell migration in inflammatory and immune responses. At the same time, GNAI2 deficiency leads to a significant defect in chemokine receptor signaling and lymphocyte transport in T cells, and GNAI2 significantly affects T cell motility in lymph nodes [51]. Proteasome Activator Subunit 1(PSME1) is an important proteasome component that is universally expressed in eukaryotic cells. Its functions involve the degradation of specific proteins, as well as misfolded proteins. Meanwhile, PSME1 plays a role in immune system management [52]. PSME1 is regulated by IFNγ, as well as being an effective agonist for MHC class I antigen presentation, which mediates the epitope recognition of cytotoxic T cells and ultimately leads to T cell activation [53]. Activated T cells have been shown to promote tumor ferroptosis as a novel antitumor mechanism. CD8+ T cells downregulate the expression of SLC3A2 and SLC7A11, two subunits of the glutamate–cystine reverse transport system Xc-, by releasing IFNγ, which in turn impairs cystine uptake by tumor cells, promoting tumor cell lipid peroxidation and the onset of ferroptosis [22]. In a study by Nasri et al. [54] PSME1 protein was found to be overexpressed in CD4+ T cells in patients with polycystic ovary syndrome and involved in several pathways of cellular metabolism, particularly glycolysis and iron death pathways. It is thus clear that both GNAI2 and PSME1 are closely related to the regulation of ferroptosis and immune function in the organism, but the specific mechanisms of how GNAI2 and PSME1 are involved in the occurrence of cellular ferroptosis need to be further investigated. In our study, we found that these two genes also play an important role in IDC, and the expression levels of the two genes were directly proportional to the survival of patients. The low expression of GNAI2 in IDC may be one of the reasons for promoting tumor progression. Notably, PSME1 is overexpressed in IDC and associated with better prognoses, which we believe may be related to genes that play different roles in tumor initiation and progression. This phenomenon was mentioned in the study of Deng et al. [55]; the ferroptosis-related gene CHAC1 was down-regulated in KIRC samples, but the expression level of CHAC1 was directly proportional to the poor prognosis of clear cell renal cell carcinoma and could be an effective indicator of a poor prognosis. Therefore, the specific mechanism of PSME1 action remains to be further elucidated.

Finally, a prognostic model constructed by GNAI2 and PSME1 stratified patients into high- and low-risk groups with significant survival differences between the two groups. RS showed good performance in predicting prognoses and was an independent prognostic factor for IDC. This study provides a deeper understanding of the underlying molecular mechanisms of IDC.

Limitations in this study may need further investigation. First of all, retrospective research data from public databases may lack useful parameters that affect patient survival and ignore heterogeneity between different populations. It is necessary to conduct a prospective analysis in a multicenter cohort for further validation. Second, our omics data is based on transcriptome-level quantification, which may have an influence on the prediction of ferroptosis-process-related components at the translational level.

## 5. Conclusions

In conclusion, the ferroptosis and immune status is associated with the prognosis of IDC patients. We stratified patients based on ferroptosis and immune status, and developed a prognostic model based on two ferroptosis-immune-related genes. It showed good prognostic stratification ability in the training cohort and the verification cohorts. It might serve as a prognostic classifier for clinical decision-making regarding individualized prognostication and treatment, and follow-up scheduling.

## Data Availability

The data presented in this study are openly available in the TCGA database (https://portal.gdc.cancer.gov (accessed on 13 February 2021)); GEO datasets (https://www.geo.org/en/ (accessed on 22 March 2021)); FerrDb database (http://www.zhounan.org/ferrdb/index.html (accessed on 24 February 2021)) and TIMER2.0 (http://timer.cistrome.org/ (accessed on 15 March 2021)).

## Supplementary Materials

The following supporting information can be downloaded at: https://www.mdpi.com/article/10.3390/biom13010147/s1, Figure S1: IDC immune−landscape; Figure S2: Differences in clinical characteristics and clinical stratified survival analysis among the subtypes; Figure S3: Distribution of individual expression of immune-related genes among subtypes; Figure S4: Clinical features of FIRS; Table S1: Clinical information of train and test sets; Table S2: univariate Cox analysis reveals prognostic−related genes.
