# Supplementary material for: Combination Analysis of Ferroptosis and Immune Status Predicts Patients Survival in Breast Invasive Ductal Carcinoma
Source: Biomolecules. 2023 Jan 11;13(1):147. doi: 10.3390/biom13010147 (PMC9855618; doi:10.3390/biom13010147)
Supplement: Supplementary file 1 [file biomolecules-13-00147-s001.zip › biomolecules-2066232-supplementary.pdf]

Table S1

Clinical information of train and test sets.

	Train	Test	X^2	P-value
**Survival**			1.886	0.17
Alive	286	294		
Dead	46	34		
**Age**			0.113	0.737
<65	237	238		
>=65	95	90		
**cancer status**			0.824	0.364
Tumor Free	261	268		
With Tumor	37	30		
Unknow	34	30		
**Stage**			3.403	0.065
StageI	52	68		
StageII-IV	248	222		
Unknow	32	38		
**T**			0.115	0.734
TI-II	269	261		
TIII-IV	36	32		
Unknow	27	35		
**N**			1.471	0.225
N0	134	36		
NI-III	165	32		
Unknow	33	39		
**PR**			0.193	0.661
Negative	116	121		
Positive	206	200		
Unknow	10	7		
**ER**			0.115	0.735
Negative	87	84		
Positive	233	239		
Unknow	12	5		
**HER2**			3.524	0.061
Negative	165	175		
Positive	57	39		
Unknow	110	114		

Table S2

univariate Cox analysis reveals prognostic−related genes (p < 0.05)

id	HR	HR.95L	HR.95H	pvalue
CD74	0.75	0.59	0.94	0.01
AIF1	0.69	0.49	0.98	0.04
TYROBP	0.72	0.53	0.99	0.04
SPI1	0.69	0.50	0.95	0.02
SELPLG	0.73	0.53	0.99	0.05
HLA-DMA	0.71	0.52	0.97	0.03
HLA-DPB1	0.77	0.60	0.99	0.04
GNAI2	0.34	0.18	0.62	0.00
APOL1	0.76	0.59	0.98	0.04
IFI35	0.63	0.47	0.85	0.00
LGALS9	0.65	0.47	0.90	0.01
PSMB10	0.61	0.42	0.87	0.01
PSME1	0.56	0.37	0.83	0.00
PSMB8	0.71	0.55	0.91	0.01
APOL2	0.67	0.47	0.96	0.03
HLA-B	0.74	0.57	0.95	0.02
RHOG	0.40	0.23	0.72	0.00
HLA-DQB1	0.79	0.62	1.00	0.05
LSP1	0.73	0.54	0.99	0.04
HCST	0.69	0.50	0.95	0.02
PLAAT4	0.83	0.70	0.98	0.03
TGFB1	0.57	0.39	0.85	0.01
UBE2L6	0.66	0.49	0.90	0.01

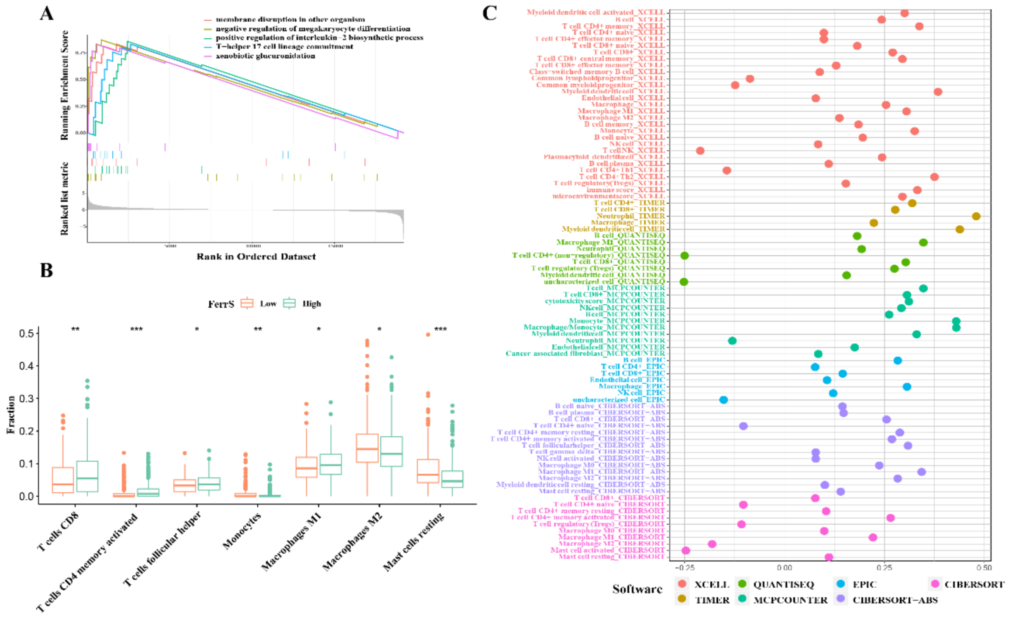

Figure S1： IDC immune−landscape: (A) GSEA GO enrichment analysis between high− and low−FerrS groups; (B) Comparison of infiltrating immune cells (CIBERSORT) between high− and low−FerrS groups. (C) The correlation between FerrS and tumor invasion results calculated by six algorithms obtained from the TIMER2 database.

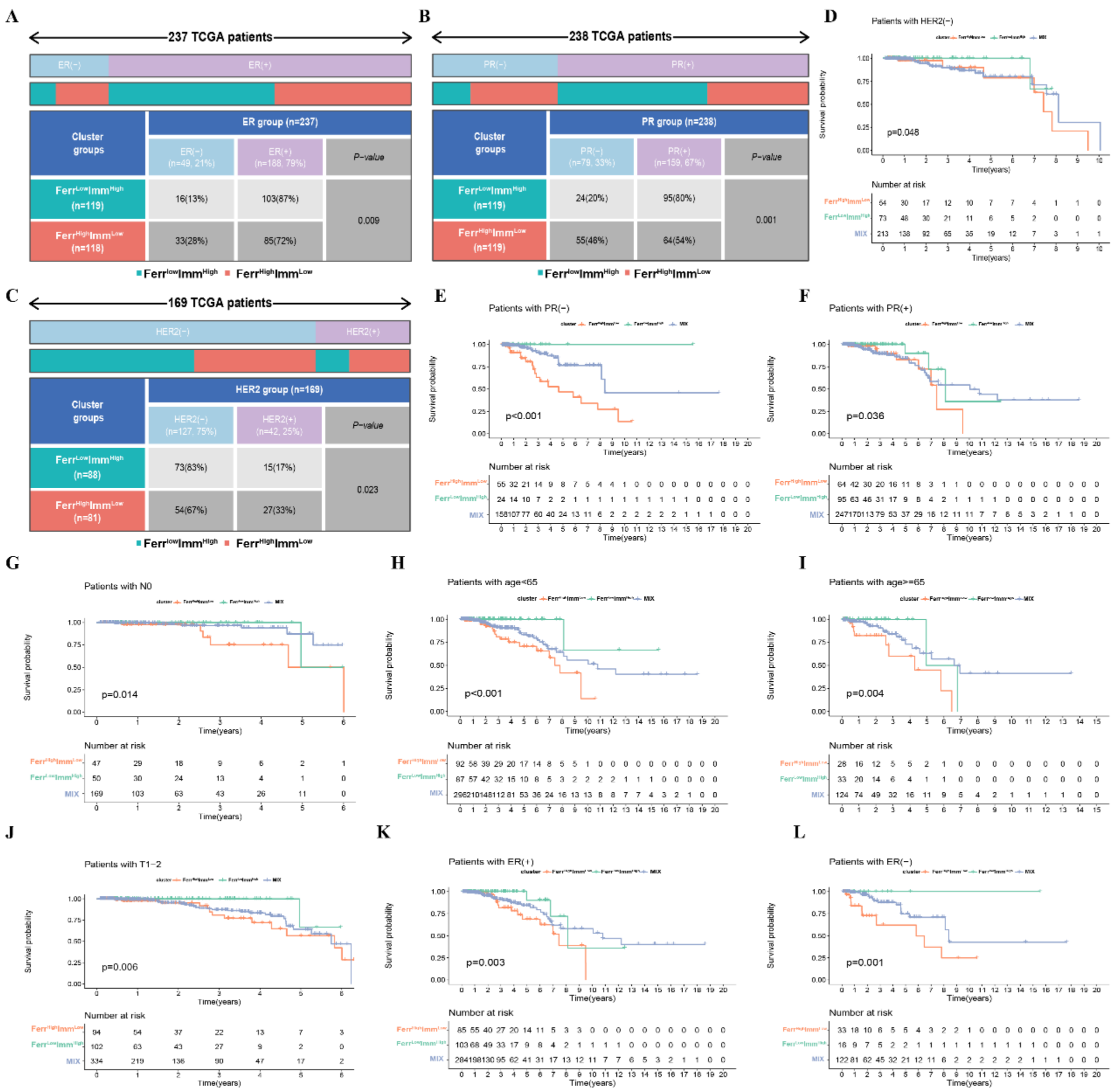

Figure S2： Differences in clinical characteristics and clinical stratified survival analysis among the subtypes: (A) The difference in ER expression between Ferr$^{\mathbf{High}}$Imm$^{\mathbf{Low}}$ group and Ferr$^{\mathbf{Low}}$Imm$^{\mathbf{High}}$ group. (B) The difference in PR expression between Ferr$^{\mathbf{High}}$Imm$^{\mathbf{Low}}$ group and Ferr$^{\mathbf{Low}}$Imm$^{\mathbf{High}}$ group. (C) The difference in HER2 expression between Ferr$^{\mathbf{High}}$Imm$^{\mathbf{Low}}$ group and Ferr$^{\mathbf{Low}}$Imm$^{\mathbf{High}}$ group. (D-L) The difference in OS between three groups stratified by clinical characteristics.

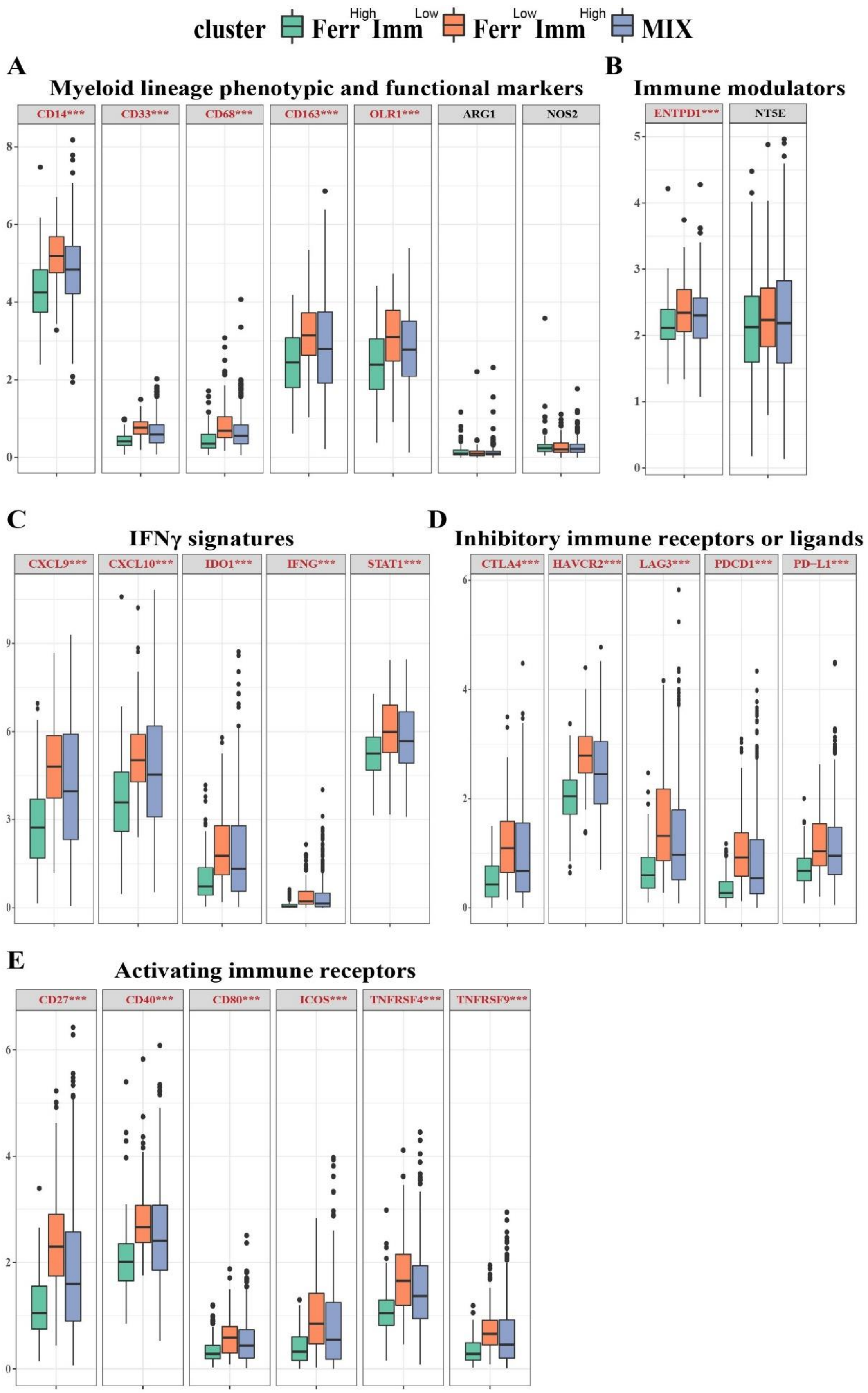

Figure S3: Distribution of individual expression of immune−related genes among subtypes. (A) the expression of myeloid lineage phenotypic and functional marker genes between three groups;

(B) the expression of immune modulator genes between three groups; (C) the expression of IFNγ marker genes between three groups; (D) the expression of immune activation receptor genes between three groups; (E) the expression of immune suppressive receptor genes between three groups;

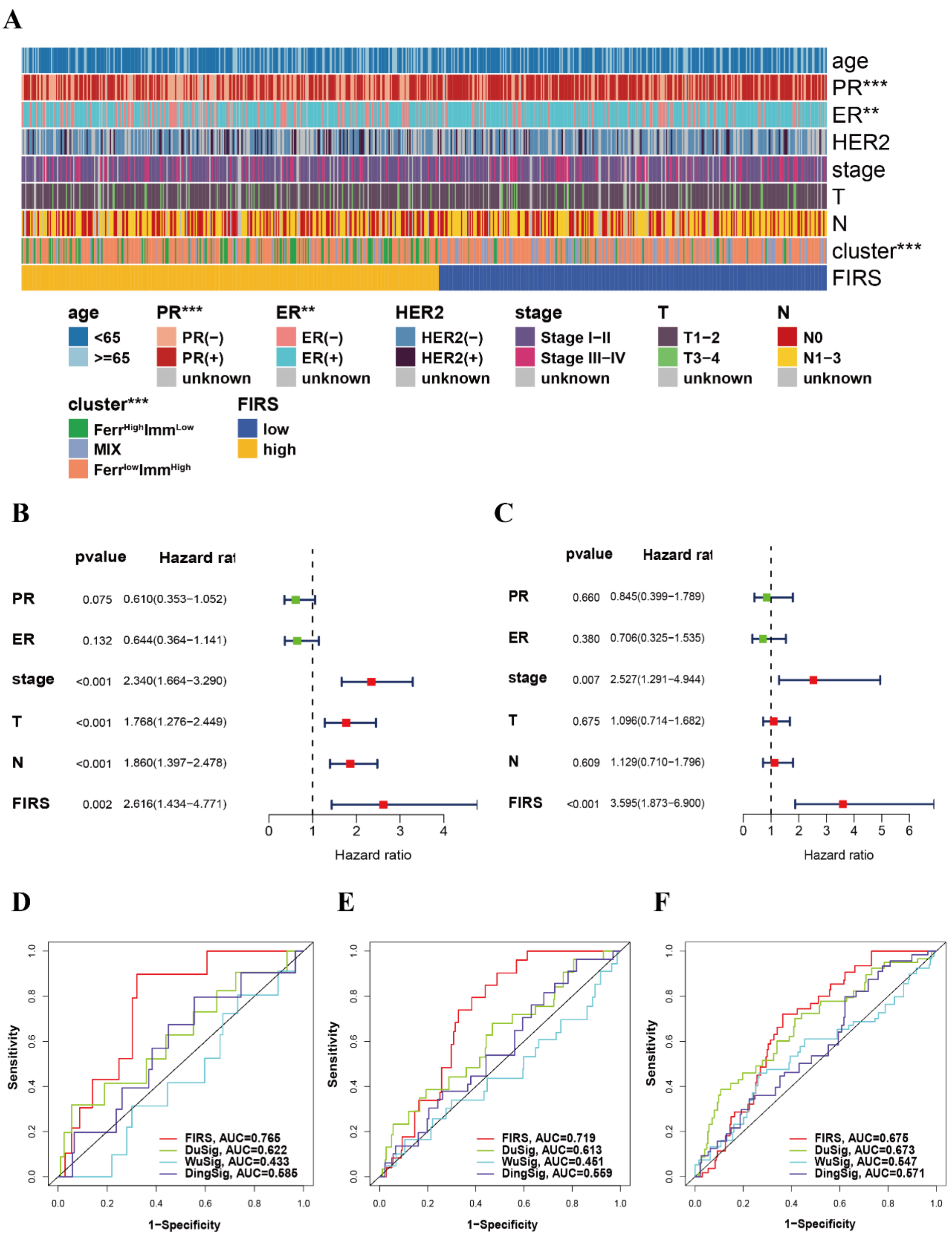

Figure S4: Clinical features of FIRS. (A) Heatmap of clinical features between subtypes. (B) Univariate COX analysis of FIRS and other clinical features. (C) Multivariate COX analysis of

FIRS and other clinical features. (D-F) Time-dependent ROC curves at 1, 2, 3 years for patients in our research and previous research.
